# Paraplégie compliquant une plaie abdominale antérieure par arme blanche

**DOI:** 10.11604/pamj.2015.20.11.5761

**Published:** 2015-01-05

**Authors:** Brahim Elahmadi, Almahdi Awab, Rachid El Moussaoui, Ahmed El Hijri, Abderrahim Azzouzi, Mustapha Alilou

**Affiliations:** 1 Service de Réanimation Chirurgicale, Hôpital Avicenne, CHU Ibn Sina, Rabat, Maroc

**Keywords:** Paraplégie, plaie abdominale, arme blanche, Paraplegia, abdominal wound, knife

## Abstract

Les traumatismes médullaires sont des complications rares des plaies abdominales antérieures par arme blanche. Son diagnostic est difficile parfois retardé. L'imagerie par résonance magnétique reste l'examen de choix. Le traitement dépend du tableau clinique et de la gravité de la souffrance médullaire. Le pronostic est corrélé à l’étendue et à la nature de la lésion médullaire. Nous rapportons un cas exceptionnel d'un traumatisme médullaire chez une patiente victime d'une plaie abdominale antérieure par arme blanche.

## Introduction

Les plaies médullaires par arme blanche sont rares. Elles constituent la troisième cause des traumatismes médullaires ouverts après les plaies par arme à feu et les plaies par accidents de la voie publique. Leur gravité est variable, parfois sévère du fait de la possibilité de déficit neurologique à récupération incertaine [1].

## Patient et observation

**Figure 1:**
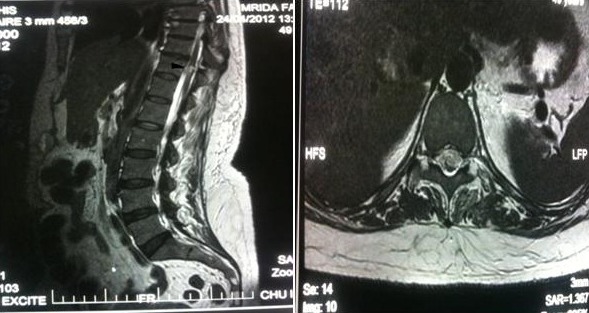
Coupes sagittale (A) et axiale (B) d'une IRM médullaire pondérées en T2 montrant une image hyper-signale transversale de la moelle en regard du D11 en rapport avec la plaie médullaire sans compression médullaire

La patiente A.M, âgée de 37 ans, sans antécédent pathologique, était victime d'une agression par coup de couteau à la région péri-ombilicale. À son admission, elle était consciente, stable sur le plan hémodynamique et respiratoire. Une plaie pénétrante de 2 cm en péri-ombilicale gauche a été constaté avec défense abdominale généralisée. L'examen neurologique a objectivé une paraplégie avec troubles sensitifs et abolition de la tonicité du sphincter anal et des reflex ostéotendineux, sans aucune plaie ou point d'impact traumatique en regard du rachis. Les pouls fémoraux étaient présents. L’échographie abdominale a objectivé un épanchement intra-péritonéal, le scanner du rachis dorsolombaire était normal. L'exploration chirurgicale abdominale a trouvé des multiples lésions grêliques. Une résection grêlique de 80 cm a été réalisé avec anastomose termino-terminale. Le bilan neuroradiologique était complété en postopératoire par une imagerie par résonance magnétique médullaire (IRM), qui a objectivé une image hyper-signale transversale de la moelle en regard de D11 sans compression médullaire (Figure 1), évoquant une section médullaire. Aucune indication chirurgicale n´était retenue. L’évolution était marquée par la persistance du déficit sensitivomoteur et des troubles sphinctériens après trois mois.

## Discussion

La grande majorité des patients victimes des plaies médullaires par arme blanche sont des adultes jeunes, de sexe masculin [2]. Le point d'impact est variable avec une prédominance de l'atteinte dorsale [3]. Il peut être aussi antérolatéral surtout au niveau cervical et exceptionnellement antérieur comme le cas présenté, expliquant la possibilité de lésions viscérales ou vasculaires associées (environ de 24%) qui aggravent le pronostic [4, 5]. Les lésions péri-rachidiennes et rachidiennes sont mineures dans les plaies par arme blanche en comparaison avec celles observées dans les plaies par arme à feu. Dans les traumatismes pénétrants, la présence d´un syndrome abdominal brouillant masque initialement le tableau neurologique et retarde la réalisation des explorations paracliniques en urgence (IRM médullaire en particulier) qui guident le protocole thérapeutique.

Sur le plan anatomopathologique, les lésions se traduisent par une section ou une compression médullaire par un hématome extradural, une esquille osseuse ou un œdème [6]. L'atteinte médullaire complète est de très mauvais pronostic, avec possibilité de récupération presque nulle. Par contre, les lésions médullaires incomplètes ont un plus fort potentiel de récupération [7].

Le scanner garde une place en l'absence de déficit neurologique en précisant la nature des dommages osseux, la trajectoire de l'arme blanche et la présence d'un pneumorachis [4]. L'IRM médullaire reste le seul examen qui permet d'identifier les lésions médullaires, et d'apprécier l'intégrité disco-ligamentaire [8]. Les indications consensuelles d'un acte opératoire urgent dans les premières heures d´une plaie médullaire sont représentées par la présence d´une fistule de liquide céphalo-rachidien ou de signes de compression et de souffrance médullaire à l'IRM [9]. Le pronostic des traumatismes médullaires dépend de l’étendue et de la nature des lésions. Des lésions minimes ou l'absence de lésions en imagerie sont en faveur d'une bonne évolution clinique, alors que les sections médullaires, les lésions contusives et hémorragiques sont grevées d'un pronostic médiocre [10].

## Conclusion

Bien que le traumatisme médullaire est une complication rare des plaies abdominales antérieures par arme blanche, le pronostic neurologique incertain fait qu'il doit être systématiquement suspecté et recherché devant la survenue de tout trouble sensitivomoteur dans ce contexte traumatique, en reconstituant le trajet même si l'orifice d'entrée est situé à distance.
